# Skin pigmentation polymorphisms associated with increased risk of melanoma in a case-control sample from southern Brazil

**DOI:** 10.1186/s12885-020-07485-x

**Published:** 2020-11-09

**Authors:** Larissa B. Reis, Renato M. Bakos, Fernanda S. L. Vianna, Gabriel S. Macedo, Vanessa C. Jacovas, André M. Ribeiro-dos-Santos, Sidney Santos, Lúcio Bakos, Patricia Ashton-Prolla

**Affiliations:** 1 Serviço de Pesquisa Experimental, Laboratório de Medicina Genômica, Hospital de Clínicas de Porto Alegre (HCPA), Rua Ramiro Barcelos, Porto Alegre, Rio Grande do Sul 2350 Brazil; 2 Programa de Pós-Graduação em Medicina: Ciências Médicas, Universidade Federal do Rio Grande do Sul (UFRGS), Porto Alegre, Brazil; 3 Serviço de Dermatologia, Hospital de Clínicas de Porto Alegre (HCPA), Porto Alegre, Brazil; 4 Programa de Pós-Graduação em Genética e Biologia Molecular, Universidade Federal do Rio Grande do Sul (UFRGS), Porto Alegre, Brazil; 5 Laboratório de Genética Humana e Médica, Universidade Federal do Pará (UFPA), Belém, Pará Brazil

**Keywords:** Melanoma, SNPs, Pigmentation

## Abstract

**Background:**

Melanoma is the most aggressive type of skin cancer and is associated with environmental and genetic risk factors. It originates in melanocytes, the pigment-producing cells. Single nucleotide polymorphisms (SNPs) in pigmentation genes have been described in melanoma risk modulation, but knowledge in the field is still limited.

**Methods:**

In a case-control approach (107 cases and 119 controls), we investigated the effect of four pigmentation gene SNPs (*TYR* rs1126809, *HERC2* rs1129038, *SLC24A5* rs1426654, and *SLC45A2* rs16891982) on melanoma risk in individuals from southern Brazil using a multivariate logistic regression model and multifactor dimensionality reduction (MDR) analysis.

**Results:**

Two SNPs were associated with an increased risk of melanoma in a dominant model: rs1129038AA and rs1426654AA [OR = 2.094 (95% CI: 1.106–3.966), *P* = 2.3 10^− 2^ and OR = 7.126 (95% CI: 1.873–27.110), *P* = 4.0 10^− 3^, respectively]. SNP rs16891982CC was associated with a lower risk to melanoma development in a log-additive model when the allele C was inherited [OR = 0.081 (95% CI: 0.008–0.782), *P* = 3 10^− 2^]. In addition, MDR analysis showed that the combination of the rs1426654AA and rs16891982GG genotypes was associated with a higher risk for melanoma (*P* = 3 10^− 3^), with a redundant effect.

**Conclusions:**

These results contribute to the current knowledge and indicate that epistatic interaction of these SNPs, with an additive or correlational effect, may be involved in modulating the risk of melanoma in individuals from a geographic region with a high incidence of the disease.

## Background

Melanoma is the most aggressive skin tumor, and its incidence has been correlated with latitude of residence, occurring most frequently in fair-skinned individuals [1]. In fact, the risk of developing melanoma diverges markedly according to skin pigmentation and geographical area, mainly due to the causative effect of ultraviolet radiation [2, 3]. Currently, Australia and New Zealand have the highest incidence and mortality rates of melanoma in the world, with incidence reaching 33.6/100,000 and 33.3/100,000, respectively [4]. In those two countries, the risk of developing melanoma before age 75 years is 1/24 and 1/34 for males and females, respectively [5]. In Brazil, melanoma represents 4% of all skin cancers, and 6260 new diagnoses are estimated in 2018. The highest incidence rates per region are expected for southern Brazil, reaching 8.4/100.000 [6]. These high rates are attributed to geographical location– the southernmost state of Brazil, Rio Grande do Sul, is within the same latitude as Australia (30.0346° South) [7]. Social practices with intense and often unprotected sun exposure and a majority-European ancestry are associated with lighter pigmentation of the skin in these individuals [7, 8].

Approximately 10% of melanomas are caused by germline mutations in cancer predisposition genes [9]. These include genes predominantly associated with melanoma (such as *CDKN2A* and *CDK4*) but also genes related to multiple solid tumors including melanoma. Examples are, among others, the *BAP1* gene, the *PTEN* gene related to Cowden’s syndrome and *XPD*, *XPC* and *XPA* genes related to Xeroderma pigmentosum. Most identifiable heritable mutations associated with hereditary melanoma have variable penetrance [10]. In addition to germline mutations, our understanding of the contribution of single nucleotide polymorphisms (SNP) proposed as risk modulators for melanoma is increasing [11]. Several common SNPs, usually of low penetrance, are commonly investigated in polygenic risk models. These models can assess the joint effect of independent SNPs in genes with lower and intermediate penetrance such as *MC1R*, *ARNT*, *CDK10*, identified by the GWAS, and can assist in the identification of individuals with a higher risk of susceptibility to melanoma [12]. Some of these SNPs are located in genes of the melanogenic pathway, and some have been described in association with melanoma in different populations across the world [13]. A complicating aspect of such studies is that, in multifactorial disorders such as melanoma, genetic and nongenetic factors, such as admixture, population substructure, and evolution patterns, can severely confound the results and result in false-positive associations [14]. Thus, differences in allele frequency between cases and controls could be associated with differences in ancestry rather than reflect an association of genes with disease [15].

The Consortium for Analysis of Diversity and Evolution in Latin America (CANDELA) is a multidisciplinary international study that involves researchers focused on studying the biological diversity of Latin Americans, analyzing samples from Mexico, Colombia, Peru, Chile, and Brazil for a wide range of issues relevant to anthropological, biological and medical research in these populations. In 2014, an analysis to evaluate a possible association between 18 SNPs in genes involved in the pigmentation pathway and Melanin Index (MI) was performed within the Brazilian cohort of Consortium for the Analysis of the Diversity and Evolution of Latin America (CANDELA) with participants born in Rio Grande do Sul (RS) and Bahia (BA), in the South and Northeast of Brazil, respectively. As a result of this analysis, four SNPs were associated with differences in MI in these populations: rs1126809 (p.Arg402Gln) on tyrosinase (*TYR*), rs1129038 (3’UTR) in the hect domain and rcc1-like domain (*HERC2*), rs1426654 (p.Thr111Ala) in solute support family 24, member 4 (*SLC24A5*) and rs16891982 (p.Phe374Leu) in the family of solute, member 2 (*SLC45A2*) carriers. Among these four SNPs, allele A of rs1426654 and allele G of rs16891982 were associated with less melanin content in the 352 participants of RS cohort (*P* < .001) [16].

In this study, we aimed to assess the association of these four SNPs with melanoma risk in southern Brazil, a region with important contribution of European ancestry and with the highest indices of melanoma in the country.

## Methods

### Samples and genotyping

This case-control study was conducted at a public University Hospital, Hospital de Clínicas de Porto Alegre (HCPA) in southern Brazil, was approved by the Institutional Review Board under number 07–139, and all participants provided informed consent. Overall, 255 unrelated individuals were recruited for the study between September 2007 and November 2008. All participants were born in the State Rio Grande do Sul, and of these, 120 had been diagnosed with melanoma. Among the melanoma patients, 19 had a family history of melanoma (melanoma in first-, second-, and/or third-degree relatives) and/or multiple primary melanomas and 101 had features of sporadic melanoma. All diagnoses were confirmed by pathology reports. The 135 individuals of the control group were recruited consecutively among patients who presented to the outpatient clinic of the same dermatology department for an initial consultation or regular follow-up for diseases other than skin cancer. None of the individuals included in the control group reported a family history of the disease, and within the patients and control groups, there were no familial relations. All patients willing to participate were clinically examined and demographic variables, pigmentation traits (eye and hair color), skin type, tanning ability, quantitative/qualitative presence of nevi, and data from primary lesions of patients with cutaneous melanoma were documented. Genomic DNA was obtained from peripheral blood, and genotyping of SNPs rs1126809 (*TYR*), rs1129038 (*HERC2*), rs1426654 (*SLC24A5*) and rs16891982 (*SLC45A2*) based on the results of the Consortium for the Analysis of the Diversity and Evolution of Latin America (CANDELA, http://www.ucl.ac.uk/silva/candela) was performed using Human Custom *TaqMan*® SNP Genotyping Assays 40X (Applied Biosystems, USA; Assay IDs: AHBKFKH; C_48033–10; C_2908190_10; C_2842665_10, respectively). Genotyping were conducted using 20 ng of genomic DNA in a *StepOneTM Real-Time PCR System* (Applied Biosystems, USA). Allelic discrimination and analysis was performed using *the Real-Time PCR software v.2.2*. The study was conducted according to the Declaration of Helsinki Principles.

### Ancestry analysis

Because the population structure due to admixture is a known confounding factor in association studies, the proportion of African, European, and Amerindian ancestry of all individuals recruited was evaluated using a previously published panel containing 61 biallelic short insertion/deletion polymorphisms (INDELs) [17].

### Statistical analysis

Genotype and allele frequencies were obtained by simple counting**.** Differences between groups were compared using Pearson’s chi square or Fisher’s exact tests. All tests were two-tailed, and significance was set at 0.05. Wilcoxon test was performed to compare ancestry profiles between cases and controls, skin types and carriers and noncarriers of fixed alleles in European populations. The study was conducted considering two scenarios: with and without population structure control.

After obtaining the proportion of African, European, and Amerindian ancestry of the individuals recruited for this study we observed that some individuals had strikingly different ancestry profiles when compared to the majority of the sample, indicating populational substructure (Additional file 1). To control for this substructure, we performed 10,000 bootstrap simulations and calculated the average 95% confidence interval of these simulations to obtain the ancestry distribution profile. Using this confidence interval, we were able to identify samples exceeding the interval. This approach identified 29 individuals, which were removed to reduce sample substructure, which could skew the analysis. The remaining samples were used in all subsequent analyses, such as in the Hardy-Weinberg equilibrium test, logistic regression and MDR. To estimate the risk of melanoma associated with selected variants, we calculated odds ratios and their 95% confidence intervals using multivariate logistic regression analysis and controlled for the following confounders: age (discrete variable); sex; skin type according to the Fitzpatrick scale in 6 subtypes, hair color, number of nevi (more or less than 50 nevi); European and African ancestry. Eye color was not used as a confounding variable because it is a covariable of the color of skin and hair. We chose these variants for adjustment since they are established risk factors for melanoma [18]. All statistical analyses were performed using SPSS®, version 18 (IBM, USA) and R.

### Multifactor dimensionality reduction

Higher-order gene-gene interactions among the SNPs associated in the logistic regression analysis were used in a nonparametric and genetic model-free multifactor dimensionality reduction (MDR) approach (version 3.0.4.). Bivariate MDR analysis was performed to verify the contribution of each SNP in the interaction and included *HERC2* rs1129038, *SLC24A5* rs1426654, and *SLC45A2* rs16891982. The model with the highest testing balance accuracy and with major cross-validation consistency was selected as the best model. Statistical significance was determined using a 1000-fold permutation test.

## Results

### Sample

Clinical features of the 254 individuals recruited are summarized in Table 1. Most individuals were female, older than 50 years at recruitment and fair-skinned (56.8% skin types I and II). The mean age was higher in the control group and the number of nevi was higher in the case group. A trihybrid ancestry profile with predominant European contribution was observed. Although admixture in the Brazilian population is expected and significant, in our sample, a homogeneity of European ancestry and a difference of European and African ancestry profiles between cases and controls were observed (*P* = .004 and *P* = .008, respectively). Additionally, mean European ancestry in individuals with skin types I and II was different than that observed in those with skin types III, IV and V (0.946, CI 95% [0.934–0.959] versus 0.902, CI 95% [0.875–0.928], *P* = .002). Moreover, carriers of almost all fixed alleles in European populations had a different ancestry profile when compared with noncarriers (the European ancestry profile A in rs1426654 was 0.928, CI 95% [0.915–0.942] and 0.746, CI 95% [0.021–1.470] in carriers and noncarriers, respectively, *P* = .033; the European ancestry profile G in rs16891982 was 0.936, CI 95% [0.924–0.947] and 0.740, CI 95% [0.561–0.918] in carriers and noncarriers, respectively, *P* < 0.001). Among melanoma patients, the average age at diagnosis was 53.72 years (SD15.5), and 23.5% had intraepithelial tumors, with the most common histological subtype being superficial spreading melanoma (Table 2).

**Table 1:** Characteristics of samples included in this study

	Global *n* = 255 (%)	Cases *n* = 120 (%)	Controls *n* = 135 (%)	***P***
**Sex**				
Female	157 (61.6)	73 (60.8)	84 (62.2)	0.461
**Age**^**a**^				
≥ 50 years old	180 (70.6)	78 (65)	102 (75.6)	
< 50 years old	75 (29.4)	42 (35)	33 (24.4)	0.012
**Mean ages (SD)**	57.01	56.31 (15.5)	58.28 (13.7)	
**Skin type**				
I	9 (3.5)	7 (5.8)	2 (1.5)	
II	136 (53.3)	66 (55)	70 (51.8)	
III	100 (39.2)	42 (35)	58 (43)	
IV	7 (2.7)	4 (3.3)	3 (2.2)	
V	3 (1.2)	1 (0.8)	2 (1.5)	0.281
**Hair color**				
Blond	52 (20.4)	30 (25)	22 (16.3)	
Red	11 (4.3)	9 (7.5)	2 (1.5)	
Light Brown	82 (32.1)	39 (32.5)	43 (31.8)	
Dark Brown	89 (35)	32 (26.7)	57 (42.2)	
Black	21 (8.2)	10 (8.3)	11 (8.1)	0.017
**Eyes color**				
Blue	70 (28.1)	39 (33.3)	31 (23.5)	
Green	47 (18.9)	27 (23)	20 (15.1)	
Brown	131 (52.6)	51 (43.6)	80 (60.6)	
Black	1 (0.4)	0	1 (0.7)	0.074
**Number of nevi**				
≥ 50	41 (16.5)	33 (28.2)	8 (6.1)	
< 50	207 (83.5)	84 (71.8)	123 (93.9)	0.000
**Number of dysplasic nevi**				
≥ 5	17 (6.8)	15 (12.8)	2 (1.5)	
< 4	231 (93.1)	102 (87.1)	129 (98.5)	0.000
**Ancestry profile**^**b**^				
European	0.971	0.945	0.911	0.004
African	0.010	0.022	0.039	0.008
Native-American	0.013	0.032	0.048	0.388

*Abbreviation*: *SD* standard deviation

^a^Samples age at recruitment

^b^Ancestrality profiles obtained for Ancestral Informative Markers (AIMs) indels panel. Date presented as median of percentile ancestry component

**Table 2:** Clinical features of patients with melanoma and Histologic aspects of their tumors

**Mean age at diagnosis (SD)**	53.72 (15.5)
**Breslow thickness/TNM Staging**^**a**^	**n (%)**
in situ/ Tis	26 (22.6)
≤ 1.0 mm/ T1	41 (35.6)
1.01–2 mm/ T2	23 (20)
2–4 mm/ T3	14 (12.2)
> 4 mm/ T4	11 (9.6)
**Histological subtype**	
Acral lentiginous	2 (1.8)
Superficial spreading	78 (72.2)
Nodular melanoma	20 (18.5)
Lentigo maligna	8 (7.4)
**Multiple primary melanoma**	
Yes	8 (6.7)
No	112 (93.3)
**Family History of melanoma**	
Yes	19 (15.8)
No	101 (84.2)

*Abbreviations*: *SD* standard deviation, *TNM* classification of malignant tumours

^a^The Breslow thickness and TNM Staging according to National Comprehensive Cancer Network (NCCN) guidelines version 1.2011

### Population substructure control

Using the 95% confidence interval calculated for each ancestry, we detected a population substructure (mean European ancestry was 0.971, CI 95% [0.583–0.991], mean African ancestry was 0.010, CI 95% [0.002–0.206], and mean Native-American ancestry was 0.013, CI 95% [0.003–0.280], see Additional file 1A. A total of 29 samples (13 cases and 16 controls) were outside the ancestry confidence interval and were excluded from the analysis of Hardy-Weinberg equilibrium and logistic regression (see Additional file 1B) in order to control for the population substructure (the entire list of excluded samples can be found in Additional Table 1).

### Genotyping

Genotyping results are summarized in Table 3. Initially, we undertook a separate analysis of the SNP frequencies in individuals with and without a family history of melanoma in the case group and did not identify a significant difference between groups (data not shown). Therefore, we have opted to continue the additional analyses including all individuals in the case group (those with and without a family history of melanoma). Linkage disequilibrium was not observed, and only rs16891982 in *SLC45A2* did not follow Hardy-Weinberg equilibrium when considering the entire sample. After controlling for substructure, all SNPs were in Hardy-Weinberg equilibrium (α = 0.05; rs1126809 *TYR χ*^2^ = 0.089, *P* = 1; rs1129038 *HERC2 χ*^2^ = 1.361, *P* = 1; rs1426654 *SLC24A5 χ*^2^ = 0.796, *P* = 1; rs16891982 *SLC45A2 χ*^2^ = 3.182, *P* = 0.498). Further analysis showed a statistically significant difference in genotypic and allelic frequencies between cases and controls for rs1426654, rs16891982, and rs1129038. Comparisons of allelic frequencies between the main population databases and other population data of southern Brazilians are shown in Additional Table 2. Allelic frequency data reinforce similarities between South Brazilians and Europeans.

**Table 3:** Allelic and genotypic frequencies of *TYR* rs1126809*, HERC2* rs1129038*, SLC24A5* rs1426654, *SLC45A2* rs16891982 variants

Gene	Chr.	Location	Position^**a**^	SNP	MAF	Genotypes	Cases n (%)	Controls n (%)	***P***
*TYR*	11	c.1205G > A	Exonic (p.Arg402Gln)	rs1126809	A = 0.22	GG	72 (46.7)	82 (53.2)	0.660^2^
						GA	45 (48.9)	47 (51.1)	
						AA	3 (33.3)	6 (66.6)	
*HERC2*	15	c.13272 + 874C > T	Intronic (3’UTR)	rs1129038	G = 0.45	AA	51 (42.5)	35 (25.9)	**0.016**^**3**^
						GA	45 (37.5)	62 (45.9)	
						GG	24 (20)	38 (28.1)	
*SLC24A5*	15	c.331A > G	Exonic (p.Ala111Thr)	rs1426654	G = 0.06	AA	117 (97.5)	111 (82.2)	**< 0.001**^**2**^
						GA	2 (1.7)	23 (17)	
						GG	1 (0.8)	1 (0.7)	
*SLC45A2*	5	c.1122C > G	Exonic (p.Phe374Leu)	rs16891982	C = 0.14	GG	103 (85.8)	93 (68.9)	**0.002**^**2**^
						CG	16 (13.3)	32 (23.7)	
						CC	1 (0.8)	10 (7.4)	

*Abbreviations*: *Chr* chromossome, *SNP* single nucleotide polymorphism, *MAF* minor allelic frequency

^a^Position in genome and protein change according to dbSNP

^2^Fisher chi-square for associating comparing the distribuition of categorical variables in cases and controls subjects. *P* values are two-sided;

^3^Pearson chi-square for associating comparing the distribuition of categorical variables in cases and controls subjects. *P* values are two-sided;

### Genetic variants and skin pigmentation

Details from the comparative data on the associations between genotypic frequencies and skin pigmentation parameters in cases and controls are shown in Additional Table 2. With the exception of SNP *TYR* rs1126809, all the SNPs were associated with certain phenotypes. The SNP *HERC2* rs1129038 AA genotype was more frequent in individuals with light skin and eyes and blond hair in both cases and controls (*P* < .001 for all analysis). The SNP *SLC24A5* rs1426654 was also associated with lighter skin and eye color, but only in the control group (*P* = .0017). The SNP *SLC45A2* rs16891982 GG genotype was more frequent in individuals with fair skin and hair both in cases and controls (*P* > .001 and *P* = .008; *P* < .001 and *P* = .004, for cases and controls, respectively). The same genotype was associated with light eye color only among cases (*P* < .001).

### Genetic variants as risk factors for melanoma

Clinical features of the melanoma patients are summarized in Table 2. Three of the four SNPs were associated with melanoma outcome. The *HERC2* rs1129038AA and *SLC45A2* rs16891982GG genotypes and the rs1426654A allele were more frequently observed in cases than controls (*P* = .0016, *P* = .002, and *P* < 0.001, respectively). In a regression logistic model, including the following risk factors: sex, age, hair color, skin type, number of nevi, and African ancestry, these associations remained strong, suggesting that they may be independent risk factors. Odds ratios (OR) for melanoma associated with genetic effect models that were obtained before and after genetic substructure control and are shown in Table 4. The dominant model for *HERC2* rs1129038 and *SLC24A5* rs1426654 was considered the best model, and in both, an increase in OR was observed after substructure control: [OR = 2.212 (95% CI: 1.106–4.426), *P* = .025] and [OR = 13.996 (95% CI: 1.711–113.995), *P* = .014], respectively. For *SLC45A2* rs16891982, the most suitable genetic model was log-additive, showing a slight reduction in the effect modification after substructure reduction when compared to the entire sample [OR = 0.068 (95% CI: 0.007–0.692), *P* = .023] and [OR = 0.081 (95% CI: 0.008–0.782), *P* = .030].

**Table 4:** Odds ratio (OR) and Genetic Effects Models with and without Genetic Subestruture evaluation

SNP	Effect	OR^a^	IC (95%)	*P*	AUC	IC (95%)
With Genetic Substructuring, *n* = 254						
rs1126809	Dominant	1.121	0.639–1.965	0.691	0.661	0.593–0.729
	Additive	1.921	0.412–8.961	0.406	0.660	0.592–0.729
	Recessive	1.999	0.444–9.003	0.367	0.660	0.592–0.728
rs1129038	**Dominant**	**2.121**	**1.105–4.070**	**0.024**	**0.714**	**0.651–0.777**
	Additive	0.510	0.220–1.183	0.117	0.714	0.651–0.777
	Recessive	1.137	0.569–2.270	0.716	0.707	0.643–0.771
rs1426654	**Dominant**	**7.164**	**1.868–27.472**	**0.004**	**0.729**	**0.667–0.791**
	Additive	1.538	0.091–25.909	0.765	0.728	0.666–0.790
	Recessive	0.509	0.029–8.937	0.644	0.697	0.632–0.763
rs16891982	Dominant	2.188	1.077–4.443	0.030	0.687	0.621–0.754
	**Additive**	**0.081**	**0.008–0.782**	**0.030**	**0.692**	**0.626–0.758**
	Recessive	9.278	0.939–91.701	0.057	0.665	0.598–0.733
Without Genetic Substructuring. *n* = 225						
rs1126809	Dominant	1.180	0.645–2.157	0.591	0.682	0.611–0.753
	Additive	3.077	0.502–18.849	0.188	0.688	0.618–0.758
	Recessive	2.951	0.494–17.643	0.235	0.689	0.619–0.759
rs1129038	**Dominant**	**2.212**	**1.106–4.426**	**0.025**	**0.705**	**0.635–0.774**
	Additive	0.390	0.157–0.967	0.042	0.698	0.629–0.767
	Recessive	1.501	0.715–3.151	0.283	0.695	0.625–0.765
rs1426654	**Dominant**	**13.966**	**1.711–113.995**	**0.014**	**0.725**	**0.659–0.790**
	Additive	0.000	0.000	0.998	0.713	0.659–0.788
	Recessive	0.000	0.000	1.000	0.693	0.623–0.762
rs16891982	Dominant	2.624	1.220–5.643	0.014	0.708	0.639–0.776
	**Additive**	**0.084**	**0.008–0.872**	**0.038**	**0.711**	**0.643–0.779**
	Recessive	9.261	0.902–95.118	0.061	0.688	0.618–0.757

*Abbreviations*: *SNP* single nucleotide polymorphism, *OR* odds ratio, *IC* confidence interval

^a^OR values was adjusted to the following risk factors: sex. Age. hair color. Skin type. Number of nevi. and African ancestry

**Fig. 1:**
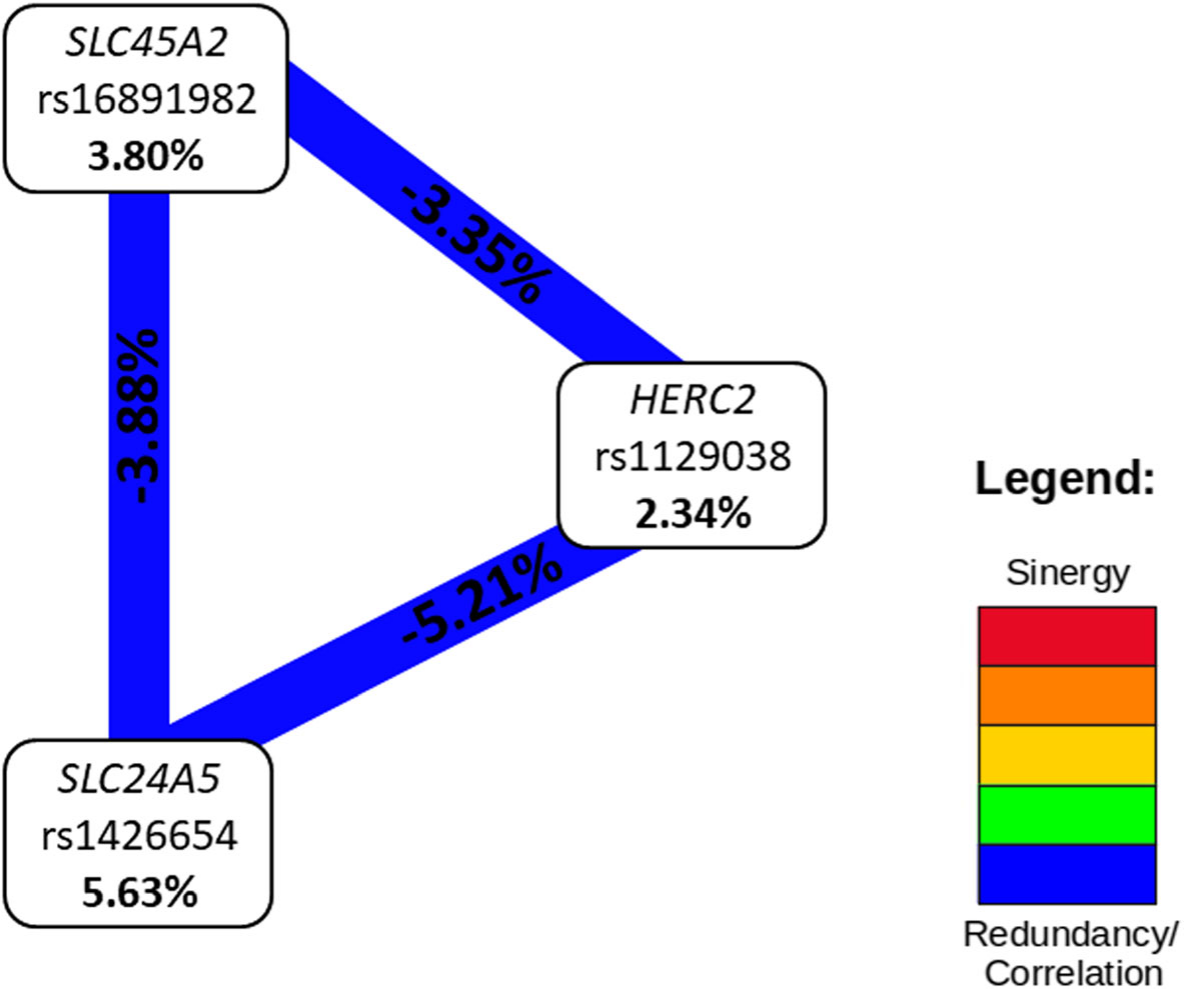
Multifactor dimensionality reduction (MDR) interaction models. Interaction circle graph comprised of nodes with pairwise connections. Values in nodes represent information gain (IG) of individual genes. While values between nodes are the IG of each pairwise combination. The type of interaction is showed by color of the line. The blue line represents negative entropy. Redundancy or linkage disequilibrium

**Fig. 2:**
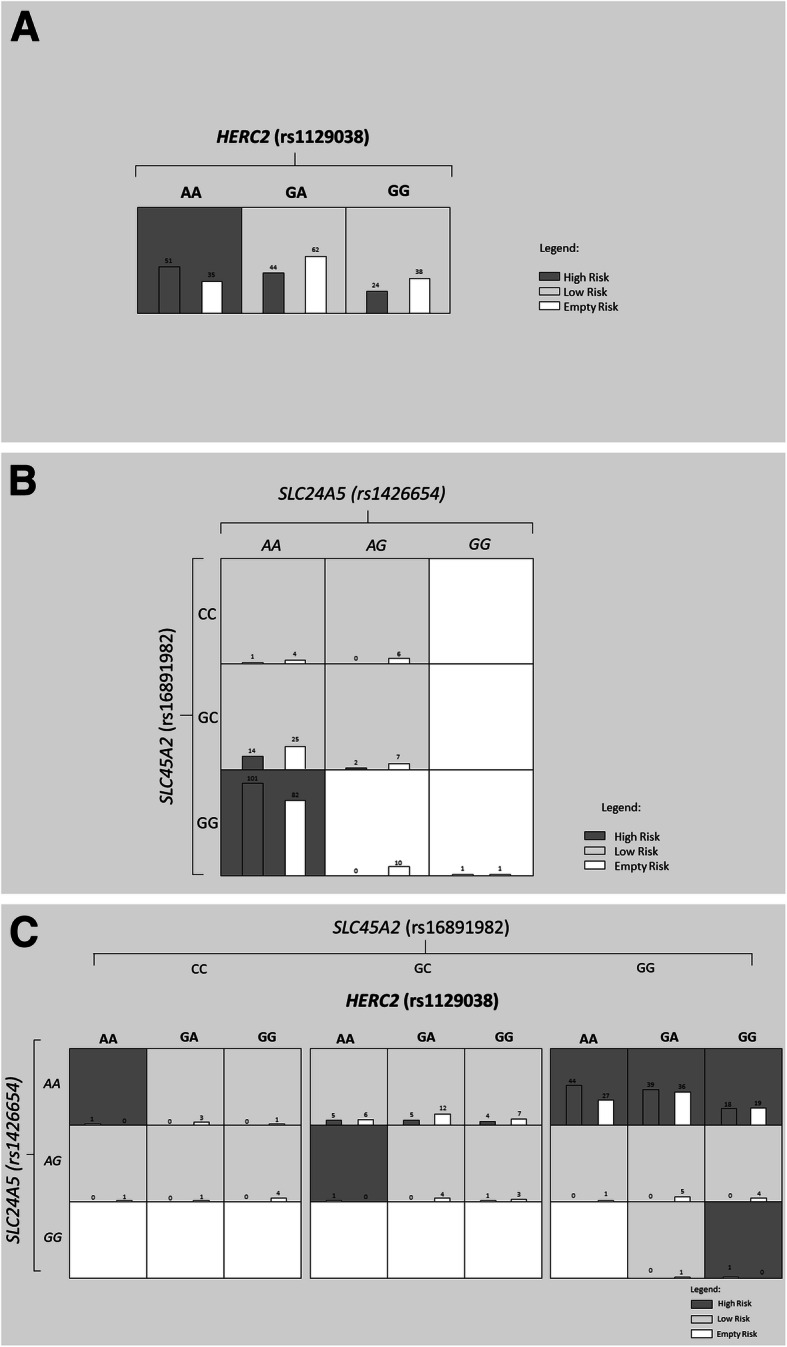
The SNPs interaction to risk with melanoma development. A high-frequency genotype combination is displayed in Dark Square. while low-frequency combinations are in lightly shaded. For each cell. The left bar indicates the absolute number of cases and the right bar the absolute number of controls. **a** The effect of *HERC2* rs1129038AA genotype. **b** The combination effect of *SLC24A5* rs1426654AA and *SLC45A2* rs16891982GG genotypes. **c** The combination effect of *SLC24A5* rs1426654AA. *SLC45A2* rs16891982GG. and *HERC2* rs1129038AA genotypes

Additionally, we performed nonparametric Multifactor dimensionality reduction (MDR, v. 3.0.4.) to detect and characterize gene-gene interactions among *HERC2* (rs1129038), *SLC24A5* (rs1426654), and *SLC45A2* (rs16891982) in risk of developing melanoma (Moore et al., 2006). Significant two- (*P* < .001) and three-locus interactions (*P* = .031) were identified in our analysis (Table 5). According to these results, the best model for predicting melanoma development was the combination of the three factors (*HERC2* rs1129038, *SLC24A5* rs1426654, and *SLC45A2* rs16891982). More details about the criteria for selecting the best model can be found in [19]. The largest main effect with the higher information gain (IG) was observed for *SLC24A5* rs1426654 (5.63%), with 100% accuracy for this model. The contribution of the other two markers, *SLC45A2* rs16891982 (3.80%) and *HERC2* rs1129038 (2.34%), indicated that they also have an important role in predicting melanoma risk. This interaction represents a high redundancy effect, which can be interpreted as an additive or correlation effect (Fig. 1). These finding are confirmed when analyzing the interaction graph with genotypic associations (Fig. 2). The combination of *SLC24A5* rs1426654AA and *SLC45A2* rs16891982GG was significantly associated with melanoma risk regardless of the genotype of *HERC2* rs1129038, confirming the lower weight of *HERC2* (2.34%) in the analysis of gene interaction. However, even isolated *HERC2* rs1129038 analysis shows that the AA genotype confers an increased risk for melanoma. Logistic regression models were created with the risk genotypes pointed out by MDR for rs1426654 and rs16891982 (AA and GG genotypes, respectively) and compared to a model without these risk alleles to identify the best model. The comparison, using ROC curve analysis, demonstrated that the model with the risk alleles is more appropriate than the model without these alleles (AUC 0.702, 95% CI 0.637–0.766 versus AUC 0.669, 95% CI 0.602–0.736).

**Table 5:** SNPs interaction by the multifactor dimensionality reduction (MDR) analysis

Models	Balanced Accuracy CV Training	Balanced Accuracy CV Testing	Cross-validation consistency	***P***^**1**^
rs1129038	0.5900	0.5190	5/10	0.564
rs1426654 and rs16891982	0.6216	0.6170	10/10	**< 0.001**
rs1129038. rs1426654 and rs16891982	0.6347	0.5863	10/10	**0.031**

^1^Evaluated using a 1000-fold permutation test to compare observed testing accuracies with those expected under the null hypothesis of null association

## Discussion

Based on a study developed by CANDELA, we investigated 4 variants previously associated with skin pigmentation in southern Brazil in melanoma patients and unaffected controls from the same geographic region [16]. A model of logistic regression including ancestry, melanoma risk factors and SNPs *HERC2* rs1129038 and *SLC24A5* rs1426654 in dominant models of inheritance showed significant associations with melanoma. The *SLC45A2* rs16891982CC SNP, in a log-additive model, was associated with a lower risk for developing the disease.

Pigmentation is a polygenic trait. and different variants have been associated with melanin levels in populations over the world [20]. One of the four SNPs investigated rs1129038, occurs in the untranslated region of *HERC2*, and three. *TYR* rs1126809, *SLC24A5* rs1426654, and *SLC45A2* rs16891982, are present in genes involved in the synthesis of melanosomes, the vesicles where melanin production and deposition occurs. In fact, rs1426654 and rs16891982 polymorphisms are determinants of pigmentation in Europeans [21], as well as in other populations [20, 22]. Our findings corroborate the association of some genotypes with lighter pigmentation and predominant European ancestry. The association of European ancestry and fair skin, eyes and hair was previously demonstrated in a sample of 1594 individuals from the same geographic region of the present study [23]. and the different ancestry profiles between darker and lighter individuals has also been previously reported [24]. Although our sample is not representative of the tri-hybrid pattern seen in most Brazilians [25], it reflects the massive colonization by Europeans in the specific region of the study [16, 23]. Just as there are regional differences in the proportions of ancestral populations in Brazil, we also expect heterogeneity in the frequency of genetic variants of specific genes, especially those related to skin, eye and hair pigmentation. Although research with admixed populations can be useful for allele detection involved in susceptibility to common diseases, the population substructure is a potential bias and should be controlled [26, 27]. In addition to a limitation in sampling (the sample was partially paired), the Hardy-Weinberg deviation found in our allelic frequencies for *SLC45A2* rs16891982 can also be explained by the occurrence of interethnic mix and population substructure. Previously identified in Europe and in other Brazilian populations [28].

The nonsynonymous SNP *TYR* rs1126809 (p.Arg402Gln) has been previously associated with light pigmentation of skin and is frequent in Caucasians. Its presence results in the reduction of activity of tyrosinase, a key enzyme in of the melanin production pathway, and some authors reported an increased risk of melanoma in carriers. Both in Europe and Australia [29]. However, we did not observe a consistent association of this SNP with pigmentation nor with risk for melanoma in our series.

On other hand, *HERC2* rs1129038, which was previously associated with lighter eye pigmentation in European populations [30, 31], showed a significantly association with fair skin. Eyes and hair in our sample. Forensic associations have described this SNP as a good predictor of blue eyes in Europeans [30, 32] and Brazilians [33]. and our findings reinforce these predictions.

Finally, SNPs *SLC24A5* rs1426654 and *SLC45A2* rs16891982 were associated with fair skin, eyes, and hair and with melanoma. *SLC24A5* rs1426654 (p.Thr111Ala) was first described in zebrafish as responsible for the golden phenotype due to a delay in melanin production during embryonic development [34]. In melanocyte cultures, homozygous GG leads to an increase in *SLC24A5* gene transcripts and a consequent increase in tyrosinase activity and melanin production [35]. The decrease of G allele frequency is gradual from Africa to Europe, indicating that a selection pressure in favor of the A allele acted on the determination of fair skin in places where the intensity of UV radiation is lower [36, 37]. Evidence of natural selection makes this SNP a frequent component of ancestral and forensic informative panels [38]. In our study, we confirmed the association of the AA genotype with fair skin and light eyes. and we identified allelic frequencies consistent with those observed in European populations [3] and in previous studies of Brazilians from other regions [39]. Likewise, SNP *SLC45A2* rs16891982 is also widely studied regarding its relationship with pigmentation in different populations. *SLC45A2* encodes the membrane-associated transporter protein carrier involved in melanin synthesis, and experimental studies in zebrafish. Mice and yeast have clearly demonstrated that the presence of the missense variant rs16891982 (p.Phe374Leu) results in decreased protein activity [40]. This SNP is also considered an ancestry informative marker (AIM), since it is able to differentiate European populations due to G allele frequency. Which is similar to the rs1426654 A allele [41]. These findings are aligned with the theory of vitamin D synthesis. Which proposes that light skin is a feature selected to compensate for the lower solar incidence in populations living far from the Equator [42] and with increased ability of the skin to respond to ultra violet (UV) radiation [43].

Alleles associated with lighter pigmentation were also associated with melanoma in our study, and this result remained significant after analysis with multivariate logistic regression adjusted for the risk factors ancestry, gender, age, eye, hair, and skin color, and number of Nevi. We considered this analysis essential to identify whether the variants studied could be considered independent risk factors for the occurrence of melanoma. Thus, SNPs *HERC2* rs1129038 and *SLC24A5* rs1426654 remained strongly associated with risk for the development of melanoma in a dominant model. The presence of homozygous genotypes of either SNP (AA for rs1426654 and AA for rs1129038) were associated with increased melanoma risk, while the *SLC45A2* rs16891982 C allele was associated with protection for melanoma as shown previously in a GWAS study in Greece composed of 284 patients and 284 controls (OR = 0.51. 95% CI 0.34–0.76; *P* = 0.001) [44]. On the other hand, an Australian sample with individuals of 100% Northern European ancestry (1.062 cases and 1.262 controls) showed the same allele associated with the risk to melanoma in logistic regression models including pigmentation features and ancestry, similar to the one presented here (OR = 2.04. 95% CI 1.27–3.40) [45] The results remained unchanged after population substructure analysis. Furthermore, the independent effects of each of these SNP were also accessed by MDR analysis, and the analysis considering the entire sample showed a redundancy interaction between the same SNPs that displayed significance through logistic regression (*P* = .031). The interaction illustrated in Fig. 1 shows that these three genes act redundantly to increase the risk of melanoma. The genotypic combinations *SLC24A5* rs1426654AA and *SLC45A2* rs16891982GG present a greater contribution in determining the risk for the disease, presenting a possible epistatic effect similar to that found between *SLC45A2* and *VDR* [46], *SLC45A2* and *OCA2*. and *MC1R* and *SLC24A5* [45].

The two most important limitations of our study are sampling process (individuals showing mostly Euro descendant ancestry in the entire sample) and relatively limited sample size. However, despite these limitations, our results are in line with previous studies and demonstrate that SNPs in genes related to pigmentation confer an independent increase in the risk for developing melanoma. In determining complex human traits in general, common genetic variants tend to have small effect sizes individually, but together. They may reveal important information and contribute to the assessment of individual risk for complex diseases such as cancer [47]. The development and evaluation of predictive models that combine environmental and genomic risk factors can help improve melanoma prevention and population screening by motivating risk reduction behaviors, especially in regions with high incidence rates. High UV radiation exposure and predominantly European ancestry [48].

Additional studies should be performed to verify whether the same scenario occurs in other regions of Brazil and Latin America. Although an association between *SLC24A5* rs1426654 and *SLC45A2* rs16891982 and melanoma has been previously described in Europeans, to our knowledge, this is the first study that confirms this association in a South American high-risk population.

## Conclusions

In this case-control study conducted in Southern Brazil, SNPs *SLC24A5* rs1426654 and *SLC45A2* rs16891982 were associated with an increased risk for melanoma, which was found to be additive and independent of pigmentation profile. These results contribute to the current knowledge about melanoma risk factors in individuals from a geographic region with a high incidence of the disease.

## Supplementary information

**Additional file 1.** Ancestry profile of samples (A) Individual European. African. and Native American ancestry inferred from 61 ancestry-informative markers in our all sample. Patients (green) and controls (orange) were compared with individuals from the putative parental populations used to infer admixture: Europeans. African. and Native Americans. (b) Individual European. African. and Native American ancestry inferred from 61 ancestry-informative markers in sample after substructure reduction. Patients (green) and controls (orange) were compared with individuals from the putative parental populations used to infer admixture: Europeans. African. and Native Americans. Admixture was estimated using STRUCTURE V.2.3.4 software.

**Additional file 2: Additional Table 1.** Samples excluded in order to reduce Population Substructure. **Additional Table 2.** Allelic and genotipic frequencies of SNPs *TYR* rs1126809, *HERC2* rs1129038, *SLC24A5* rs1426654, and *SLC45A2* rs16891982 in Southern Brazil samples and in main populacional databases

## Data Availability

Not applicable.

## Abbreviations

SNP: Single nucleotide polymorphisms
MDR: Multifactor dimensionality reduction
CANDELA: Consortium for Analysis of Diversity and Evolution in Latin America
MI: Melanin Index
RS: Rio Grande do Sul
BA: Bahia
HCPA: Hospital de Clínicas de Porto Alegre
CI: Interval confidence
OR: Odds ratio
IG: Information gain
AIM: Ancestry informative marker
UV: Ultravioleta radiation
