# Supplementary figures and images for: Skin pigmentation polymorphisms associated with increased risk of melanoma in a case-control sample from southern Brazil
Source: BMC Cancer. 2020 Nov 9;20:1069. doi: 10.1186/s12885-020-07485-x (PMC7650158; doi:10.1186/s12885-020-07485-x)

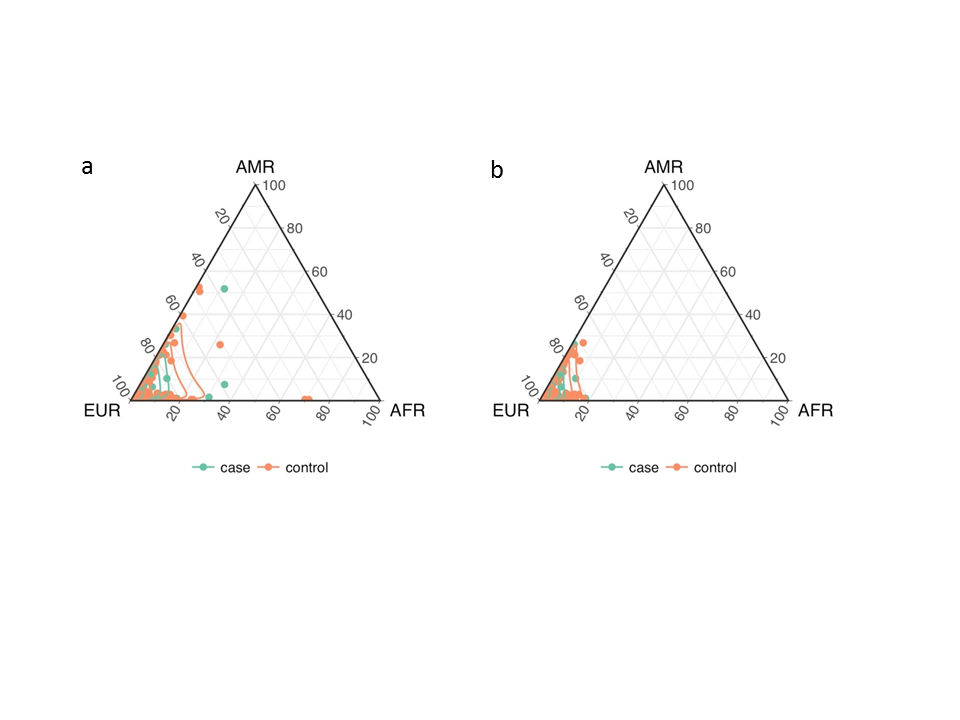

Supplement: Supplementary file 1 — Additional file 1. Ancestry profile of samples (A) Individual European. African. and Native American ancestry inferred from 61 ancestry-informative markers in our all sample. Patients (green) and controls (orange) were compared with individuals from the putative parental populations used to infer admixture: Europeans. African. and Native Americans. (b) Individual European. African. and Native American ancestry inferred from 61 ancestry-informative markers in sample after substructure reduction. Patients (green) and controls (orange) were compared with individuals from the putative parental populations used to infer admixture: Europeans. African. and Native Americans. Admixture was estimated using STRUCTURE V.2.3.4 software. [file 12885_2020_7485_MOESM1_ESM.zip › Additional File 1R4.tif]
