# Supplementary material for: Skin pigmentation polymorphisms associated with increased risk of melanoma in a case-control sample from southern Brazil
Source: BMC Cancer. 2020 Nov 9;20:1069. doi: 10.1186/s12885-020-07485-x (PMC7650158; doi:10.1186/s12885-020-07485-x)
Supplement: Supplementary file 2 — Additional file 2: Additional Table 1. Samples excluded in order to reduce Population Substructure. Additional Table 2. Allelic and genotipic frequencies of SNPs TYR rs1126809, HERC2 rs1129038, SLC24A5 rs1426654, and SLC45A2 rs16891982 in Southern Brazil samples and in main populacional databases [file 12885_2020_7485_MOESM2_ESM.docx]

**Additional Table 1 – Samples excluded in order to reduce Population Substructure**

| ID | European | African | Native-American | status | Skin type | Hair | Eyes | rs1126809 | rs129038 | rs1426654 | rs16891982 | Age |
| --- | --- | --- | --- | --- | --- | --- | --- | --- | --- | --- | --- | --- |
| 15 | 0.678 | 0.306 | 0.016 | case | II | black | brown | GG | AA | AA | GG | >50 |
| 21 | 0.990 | 0.002 | 0.007 | case | I | red | blue | GG | GG | AA | GG | <50 |
| 23 | 0.365 | 0.117 | 0.518 | case | I | blonde | blue | GG | GG | AA | GC | >50 |
| 31 | 0.586 | 0.339 | 0.075 | case | II | light brown | brown | GG | AA | AG | GC | >50 |
| 41 | 0.983 | 0.014 | 0.003 | case | II | blonde | brown | AG | AG | AA | GG | <50 |
| 47 | 0.988 | 0.009 | 0.003 | case | I | black | blue | AG | GG | AA | GG | >50 |
| 58 | 0.652 | 0.015 | 0.333 | case | II | dark brown | green | AG | GG | AG | GC | >50 |
| 63 | 0.991 | 0.002 | 0.006 | case | I | black | green | AA | AA | AA | GG | >50 |
| 73 | 0.746 | 0.248 | 0.005 | control | I | blonde | grey | GG | AG | AG | GG | <50 |
| 75 | 0.470 | 0.024 | 0.505 | control | II | light brown | brown | GG | AG | AA | GC | >50 |
| 85 | 0.594 | 0.013 | 0.393 | control | II | dark brown | brown | GG | AA | AA | GC | <50 |
| 102 | 0.992 | 0.003 | 0.005 | control | II | dark brown | blue | GG | GG | AA | GG | >50 |
| 108 | 0.298 | 0.695 | 0.006 | control | IV | light brown | brown | GG | GG | AG | CC | >50 |
| 117 | 0.988 | 0.002 | 0.010 | control | III | black | blue | AG | GG | AA | GG | >50 |
| 123 | 0.922 | 0.004 | 0.004 | case | I | dark brown | brown | GG | AA | AA | GG | <50 |
| 124 | 0.922 | 0.005 | 0.003 | case | III | black | brown | AG | AG | AA | GG | <50 |
| 133 | 0.992 | 0.005 | 0.004 | case | II | blonde | blue | AG | GG | AA | GG | >50 |
| 136 | 0.922 | 0.003 | 0.005 | case | III | dark brown | blue | GG | GG | AA | GG | >50 |
| 148 | 0.944 | 0.003 | 0.003 | case | III | black | grey | GG | AG | AA | GG | >50 |
| 186 | 0.989 | 0.007 | 0.003 | control | III | black | blue | GG | AG | AA | GG | >50 |
| 189 | 0.689 | 0.009 | 0.303 | control | III | black | brown | GG | AG | GG | GG | >50 |
| 204 | 0.755 | 0.240 | 0.006 | control | II | blonde | blue | AG | GG | AA | GG | >50 |
| 213 | 0.283 | 0.712 | 0.005 | control | II | dark brown | brown | GG | AA | AG | CC | <50 |
| 236 | 0.991 | 0.006 | 0.003 | control | III | dark brown | brown | GG | AG | AA | GG | >50 |
| 239 | 0.853 | 0.144 | 0.003 | control | III | black | brown | AG | AG | AA | GG | <50 |
| 251 | 0.994 | 0.003 | 0.003 | control | III | dark brown | blue | GG | GG | AA | GG | <50 |
| 262 | 0.512 | 0.229 | 0.259 | control | II | light brown | brown | GG | AG | AG | GG | >50 |
| 268 | 0.989 | 0.009 | 0.009 | control | II | dark brown | green | GG | AG | AA | GG | >50 |
| 271 | 0.463 | 0.526 | 0.526 | control | III | dark brown | brown | GG | AG | AG | CC | >50 |

## Additional Table 2

| **Additional Table 2. Allelic and genotipic frequencies of SNPs *TYR*  rs1126809, *HERC2* rs1129038, *SLC24A5* rs1426654, and *SLC45A2* rs16891982 in Southern Brazil samples and in main populacional databases** | | | | | | | | | |
| --- | --- | --- | --- | --- | --- | --- | --- | --- | --- |
|  |  |  |  |  |  |  |  |  |  |
|  | **Reis, LB et al., 2018** | **Cerqueira et al., 2014** | **ABraOM** | **1000genomas** | | | **ExAC** | | |
|  | "Gauchos" | "Gauchos" | Brazilians | Europeans | Africans | Americans | Europeans | Africans | Latin |
| *TYR* rs1126809 | | | | | | | | | |
| G | 0.78 | 0.81 | 0.853 | 0.748 | 0.991 | 0.875 | 0.736 | 0.954 | 0.909 |
| A | 0.22 | 0.19 | 0.146 | 0.252 | 0.009 | 0.125 | 0.264 | 0.046 | 0.091 |
| GG | 0.61 | 0.66 | 0.727 | 0.545 | 0.985 | 0.761 | 0.542 | 0.910 | 0.826 |
| GA | 0.34 | 0.28 | 0.249 | 0.406 | 0.012 | 0.228 | 0.388 | 0.087 | 0.165 |
| AA | 0.05 | 0.04 | 0.021 | 0.050 | 0.003 | 0.012 | 0.069 | 0.002 | 0.008 |
| *HERC2* rs1129038 | | | | | | | | | |
| G | 0.452 | 0.56 | 0.260 | 0.365 | 0.972 | 0.797 | 0.253 | 0.879 | 0.854 |
| A | 0.546 | 0.44 | 0.740 | 0.635 | 0.028 | 0.203 | 0.747 | 0.121 | 0.146 |
| GG | 0.243 | 0.32 | 0.006 | 0.177 | 0.947 | 0.651 | 0.067 | 0.772 | 0.739 |
| GA | 0.419 | 0.47 | 0.384 | 0.376 | 0.050 | 0.291 | 0.384 | 0.212 | 0.240 |
| AA | 0.337 | 0.20 | 0.547 | 0.447 | 0.003 | 0.058 | 0.547 | 0.014 | 0.019 |
| *SLC24A5* rs1426654 | | | | | | | | | |
| A | 0.94 | 0.90 | 0.751 | 0.997 | 0.074 | 0.589 | 0.996 | 0.199 | 0.448 |
| G | 0.06 | 0.10 | 0.249 | 0.003 | 0.926 | 0.411 | 0.004 | 0.801 | 0.562 |
| AA | 0.89 | 0.83 | 0.564 | 0.994 | 0.008 | 0.401 | 0.992016 | 0.039 | 0.193 |
| AG | 0.10 | 0.15 | 0.373 | 0.006 | 0.133 | 0.378 | 0.007968 | 0.318 | 0.492 |
| GG | 0.01 | 0.02 | 0.062 | 0 | 0.859 | 0.222 | 0.000016 | 0.641 | 0.313 |
| *SLC45A2* rs16891982 | | | | | | | | | |
| C | 0.14 | 0.23 | 0.407 | 0.062 | 0.964 | 0.536 | 0.048 | 0.846 | 0.661 |
| G | 0.86 | 0.77 | 0.593 | 0.938 | 0.036 | 0.464 | 0.952 | 0.154 | 0.339 |
| CC | 0.04 | 0.06 | 0.165 | 0.008 | 0.933 | 0.354 | 0.002 | 0.715 | 0.435 |
| CG | 0.19 | 0.32 | 0.482 | 0.107 | 0.062 | 0.363 | 0.095 | 0.260 | 0.448 |
| GG | 0.77 | 0.60 | 0.351 | 0.885 | 0.005 | 0.282 | 0.902 | 0.023 | 0.115 |
| Abbreviations: ABraOM, Online Archive of Brazilian Mutations; ExAc, Exome Aggregation Consortium. | | | | | | | | | |
